# Supplementary material for: Physical/Chemical Properties and Resorption Behavior of a Newly Developed Ca/P/S-Based Bone Substitute Material
Source: Materials (Basel). 2020 Aug 5;13(16):3458. doi: 10.3390/ma13163458 (PMC7475886; doi:10.3390/ma13163458)
Supplement: Supplementary file 1 [file materials-13-03458-s001.pdf]

materials

Supplementary

Physical/Chemical Properties and Resorption Behavior of a Newly Developed Ca/P/S-Based Bone Substitute Material

Bing-Chen Yang [1], Jing-Wei Lee [2], Chien-Ping Ju [1,*] and Jiin-Huey Chern Lin [1,*]

[1] Department of Materials Science and Engineering, College of Engineering, National Cheng Kung University, Tainan 70101, Taiwan; N58011308@ncku.edu.tw

[2] Division of Plastic and Reconstructive Surgery, National Cheng Kung University Hospital, Department of Surgery, College of Medicine, National Cheng Kung University, Tainan 007A 70403, Taiwan; jwlee@mail.ncku.edu.tw

* Correspondence: cpju@mail.ncku.edu.tw (C.-P.J.); chernlin@mail.ncku.edu.tw (J.-H.C.L.); Tel.: +886-6-2748086 (C.-P.J.); +886-6-2080391 (J.-H.C.L.)

Received: 3 July 2020; Accepted: 2 August 2020; Published: date

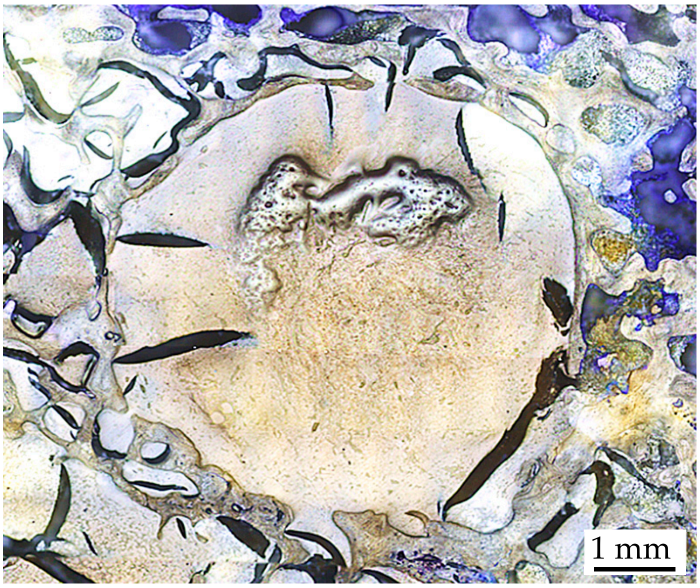

Figure S1. TB-stained superimposed composite image of NZW rabbit femur condyle in negative control at 12 weeks post-operation.

Table S1. Semi-quantitatively-determined phase contents (wt%) of CBS-400.

Phase / Sample#	HA	CSD	TTCP	DCPA
1	43.43	27.22	10.57	18.78
2	40.49	23.62	19.16	16.73
3	33.15	38.44	9.46	18.95
4	43.20	26.81	9.75	20.24
5	43.01	23.46	15.32	18.21
6	40.82	32.18	10.72	16.28
Average ± SD	40.68 ± 3.90.	28.62 ± 5.76	12.50 ± 3.90	18.20 ± 1.48

Table S2. Normalized compositions (at%) of CBS-400 determined by SEM/EDS.

**Sample #**	**Site #**	**Ca**	**P**	**S**
1	1	54.74	37.57	7.69
	2	54.18	38.26	7.56
	3	54.24	38.15	7.61
	4	54.21	38.11	7.69
	5	54.36	38.09	7.55
	6	53.99	38.09	7.92
2	1	54.45	38.10	7.45
	2	54.62	38.13	7.25
	3	54.35	38.52	7.14
	4	54.27	38.15	7.58
	5	54.14	38.24	7.61
	6	54.43	38.21	7.36
3	1	54.78	39.52	5.70
	2	54.40	39.83	5.77
	3	54.39	39.88	5.73
	4	54.68	39.60	5.72
	5	54.88	39.15	5.97
	6	55.14	39.41	5.45
4	1	55.67	38.89	5.44
	2	55.61	38.33	6.06
	3	55.14	39.09	5.76
	4	54.94	39.30	5.76
	5	55.11	38.79	6.10
	6	55.55	38.70	5.76
5	1	54.97	39.97	5.06
	2	54.83	40.00	5.16
	3	54.59	40.23	5.18
	4	54.53	40.41	5.06
	5	54.54	40.10	5.36
	6	54.27	40.66	5.07
6	1	54.17	40.79	5.03
	2	54.31	40.45	5.24
	3	54.57	40.08	5.35
	4	54.45	40.19	5.36
	5	54.48	40.22	5.30
	6	54.05	40.26	5.69
Average		54.61	39.21	6.18
SD		0.43	0.95	1.01
Ca/P ratio		1.39		

Table S3. Minimum Feret diameter (μm) of CBS-400 (unit: number of particles).

Sample # / Minimum Feret Diameter (μm)	1	2	3	4	5	6	Sum
0–100	0	0	0	0	0	0	0
100–200	11	44	5	27	37	77	201
200–300	9	24	13	15	24	40	125
300–400	59	70	6	20	29	47	231
400–500	269	415	22	112	113	141	1072
500–600	384	650	40	209	383	364	2030
600–700	287	443	61	249	461	376	1877
700–800	242	270	74	234	354	235	1409
800–900	196	174	108	197	272	175	1122
900–1000	140	148	110	126	169	148	841
1000–1100	105	101	96	124	123	121	670
1100–1200	97	74	106	111	89	92	569
1200–1300	75	59	88	76	77	90	465
1300–1400	42	44	64	38	52	37	277
1400–1500	18	15	15	12	23	25	108
1500–1600	3	4	3	7	18	5	40
1600–1700	1	1	3	1	5	0	11
Sum	1938	2536	814	1558	2229	1973	11048

Table S4. Pore size measurement data (number of pores in different size ranges) of CBS-400.

Pore Size Range (μm) \ Sample	1	2	3	Sum
0~20	1	0	0	1
21~40	0	0	3	3
41~60	3	1	30	34
61~80	31	39	60	130
81~100	121	140	103	364
101~120	167	154	133	454
121~140	117	102	89	308
141~160	39	42	55	136
161~180	15	16	20	51
181~200	6	6	7	19
Sum	500	500	500	1500
Average (μm)	114	113	109	112
SD (μm)	25	26	32	28

Table S5. Porosity values (vol%) of CBS-400 granular samples.

Sample #	1	2	3	4	5	6	Average	SD
CBS-400	76.88	77.03	76.57	78.85	77.78	79.91	77.84	1.30

Table S6. pH values of the daily-refreshed Hanks' solution wherein CBS-400 were immersed for 1, 2, 3, 4, 5, 6, 7 and 14 days.

Sample #	1	2	3	4	5	6	Average	SD
Day 1	5.27	5.19	5.28	5.29	5.39	5.27	5.28	0.07
Day 2	5.77	5.70	5.71	5.71	5.85	5.97	5.78	0.11
Day 3	5.79	5.83	5.72	5.83	5.91	5.99	5.85	0.10
Day 4	6.03	6.00	5.98	5.98	6.06	6.13	6.03	0.06
Day 5	6.23	6.21	6.27	6.14	6.29	6.37	6.25	0.08
Day 6	6.34	6.40	6.44	6.37	6.48	6.56	6.43	0.08
Day 7	6.50	6.51	6.57	6.54	6.55	6.63	6.55	0.05
Day 14	7.06	7.09	7.11	7.03	7.05	7.13	7.08	0.04

Table S7. Concentrations of Ca, P, and S in the TRIS-HCl and buffered citric acid solutions wherein CBS-400 were immersed.

Sample	Ca	P	S
	ppm	ppm	ppm
TRIS-HCl			
Background (TRIS-HCl)	3.220	0.063	ND
24 h-1	234.8	4.738	411.6
24 h-2	251.3	5.062	452.9
24 h-3	252.8	4.410	460.9
24 h-4	249.1	4.561	448.9
72 h-1	354.3	9.377	591.2
72 h-1	361.1	9.529	603.4
72 h-2	364.6	9.411	602.9
72 h-3	373.5	9.597	615.6
120 h-1	407.7	14.43	658.9
120 h-2	415.4	14.38	653.7
120 h-3	419.9	14.66	685.8
120 h-4	423.1	14.71	682.3
Citric acid			
Background (citric acid)	1.867	0.093	ND
120 h-1	1156	1234	489.1
120 h-2	1170	1238	489.1
120 h-3	1265	1345	520.7
120 h-4	1249	1325	532.8

Table S8. Optical density of the medium, CBS-400, Al_2O_3 and phenol groups in cytotoxicity test.

**Medium**	**CBS-400**	**CBS-400**	**CBS-400**	**CBS-400**	**Al_2O_3**	**0.3% Phenol**	**Medium**
Optical density (450 nm)							
1.094	0.842	0.798	0.915	0.998	1.078	0.513	1.121
1.118	0.935	0.881	1.013	0.861	1.047	0.486	1.132
1.159	0.879	0.938	1.085	0.849	1.133	0.462	1.33
1.138	0.915	0.997	0.849	1.098	1.093	0.648	1.099
1.131	1.074	1.051	0.953	0.911	1.137	0.558	1.262
1.136	1.027	1.043	1.007	0.973	1.085	0.593	1.133
Optical density (630 nm)							
0.069	0.044	0.057	0.051	0.054	0.051	0.052	0.052
0.071	0.061	0.066	0.064	0.065	0.056	0.054	0.055
0.058	0.052	0.055	0.057	0.056	0.051	0.056	0.054
0.061	0.069	0.065	0.061	0.063	0.071	0.054	0.053
0.055	0.055	0.055	0.055	0.055	0.065	0.065	0.056
0.051	0.059	0.058	0.06	0.059	0.063	0.063	0.061

Table S9. Scores of CBS-400 extract and blank control sites for intracutaneous (intradermal) reactions (Draize scale).

**Animal 1**	**Immediate**		**24 h**		**48 h**		**72 h**	
	**Erythema**	**Oedema**	**Erythema**	**Oedema**	**Erythema**	**Oedema**	**Erythema**	**Oedema**
Polar extract								
site 1	0	0	0	0	0	0	0	0
site 2	0	0	0	0	0	0	0	0
site 3	0	0	0	0	0	0	0	0
site 4	0	0	0	0	0	0	0	0
site 5	0	0	0	0	0	0	0	0
Polar vehicle								
site 1	0	0	0	0	0	0	0	0
site 2	0	0	0	0	0	0	0	0
site 3	0	0	0	0	0	0	0	0
site 4	0	0	0	0	0	0	0	0
site 5	0	0	0	0	0	0	0	0
Non-polar extract								
site 1	0	0	0	0	0	0	0	0
site 2	0	0	0	0	0	0	0	0
site 3	0	0	0	0	0	0	0	0
site 4	0	0	0	0	0	0	0	0
site 5	0	0	0	0	0	0	0	0
Non-polar vehicle								
site 1	0	0	0	0	0	0	0	0
site 2	0	0	0	0	0	0	0	0
site 3	0	0	0	0	0	0	0	0
site 4	0	0	0	0	0	0	0	0
site 5	0	0	0	0	0	0	0	0
Animal 2	Immediate		24 h		48 h		72 h	
	Erythema	Oedema	Erythema	Oedema	Erythema	Oedema	Erythema	Oedema
Polar extract								
site 1	0	0	0	0	0	0	0	0
site 2	0	0	0	0	0	0	0	0
site 3	0	0	0	0	0	0	0	0
site 4	0	0	0	0	0	0	0	0
site 5	0	0	0	0	0	0	0	0
Polar vehicle								
site 1	0	0	0	0	0	0	0	0
site 2	0	0	0	0	0	0	0	0
site 3	0	0	0	0	0	0	0	0
site 4	0	0	0	0	0	0	0	0
site 5	0	0	0	0	0	0	0	0
Non-polar extract								
site 1	0	0	0	0	0	0	0	0
site 2	0	0	0	0	0	0	0	0
site 3	0	0	0	0	0	0	0	0
site 4	0	0	0	0	0	0	0	0
site 5	0	0	0	0	0	0	0	0
Non-polar vehicle								
site 1	0	0	0	0	0	0	0	0
site 2	0	0	0	0	0	0	0	0
site 3	0	0	0	0	0	0	0	0
site 4	0	0	0	0	0	0	0	0

site 5	0	0	0	0	0	0	0	0
Animal 3	Immediate		24 h		48 h		72 h	
	Erythema	Oedema	Erythema	Oedema	Erythema	Oedema	Erythema	Oedema
Polar extract								
site 1	0	0	0	0	0	0	0	0
site 2	0	0	0	0	0	0	0	0
site 3	0	0	0	0	0	0	0	0
site 4	0	0	0	0	0	0	0	0
site 5	0	0	0	0	0	0	0	0
Polar vehicle								
site 1	0	0	0	0	0	0	0	0
site 2	0	0	0	0	0	0	0	0
site 3	0	0	0	0	0	0	0	0
site 4	0	0	0	0	0	0	0	0
site 5	0	0	0	0	0	0	0	0
Non-polar extract								
site 1	0	0	0	0	0	0	0	0
site 2	0	0	0	0	0	0	0	0
site 3	0	0	0	0	0	0	0	0
site 4	0	0	0	0	0	0	0	0
site 5	0	0	0	0	0	0	0	0
Non-polar vehicle								
site 1	0	0	0	0	0	0	0	0
site 2	0	0	0	0	0	0	0	0
site 3	0	0	0	0	0	0	0	0
site 4	0	0	0	0	0	0	0	0
site 5	0	0	0	0	0	0	0	0

Table S10. Scores of positive control and blank control sites for intracutaneous (intradermal) reactions (Draize scale).

**Animal 1**	**Immediate**		**24 h**		**48 h**		**72 h**	
	**Erythema**	**Oedema**	**Erythema**	**Oedema**	**Erythema**	**Oedema**	**Erythema**	**Oedema**
				Formalin				
site 1	0	0	0	1	1	3	1	2
site 2	0	0	0	1	1	1	1	1
site 3	0	0	0	1	1	2	1	1
site 4	0	0	1	0	2	1	3	1
site 5	0	0	1	0	1	1	3	1
				Polar vehicle				
site 1	0	0	0	0	0	0	0	0
site 2	0	0	0	0	0	0	0	0
site 3	0	0	0	0	0	0	0	0
site 4	0	0	0	0	0	0	0	0
site 5	0	0	0	0	0	0	0	0
				Histamine				
site 1	0	0	1	0	2	1	2	1
site 2	0	0	0	1	1	1	2	1
site 3	0	0	1	4	4	3	4	2
site 4	0	0	1	1	2	1	2	2
site 5	0	0	0	1	1	1	1	1
				Non-polar vehicle				
site 1	0	0	0	0	0	0	0	0
site 2	0	0	0	0	0	0	0	0
site 3	0	0	0	0	0	0	0	0
site 4	0	0	0	0	0	0	0	0
site 5	0	0	0	0	0	0	0	0
Animal 2	Immediate		24 h		48 h		72 h	
	Erythema	Oedema	Erythema	Oedema	Erythema	Oedema	Erythema	Oedema
				Formalin				
site 1	0	0	2	3	3	3	3	2
site 2	0	0	2	3	2	3	2	3
site 3	0	0	2	3	3	3	3	2
site 4	0	0	2	3	2	3	3	2
site 5	0	0	2	3	1	3	3	3
				Polar vehicle				
site 1	0	0	0	0	0	0	0	0
site 2	0	0	0	0	0	0	0	0
site 3	0	0	0	0	0	0	0	0
site 4	0	0	0	0	0	0	0	0
site 5	0	0	0	0	0	0	0	0
				Histamine				
site 1	0	0	1	1	1	1	1	2
site 2	0	0	1	1	1	2	1	2
site 3	0	0	1	0	1	1	3	1
site 4	0	0	0	0	1	2	1	2
site 5	0	0	0	1	1	1	1	1
				Non-polar vehicle				
site 1	0	0	0	0	0	0	0	0
site 2	0	0	0	0	0	0	0	0
site 3	0	0	0	0	0	0	0	0
site 4	0	0	0	0	0	0	0	0

site 5	0	0	0	0	0	0	0	0
Animal 3	Immediate		24 h		48 h		72 h	
	Erythema	Oedema	Erythema	Oedema	Erythema	Oedema	Erythema	Oedema
Formalin								
site 1	0	0	1	2	1	3	4	3
site 2	0	0	1	2	2	3	2	3
site 3	0	0	1	2	1	3	2	3
site 4	0	0	1	2	2	3	2	3
site 5	0	0	1	2	1	3	2	3
Polar vehicle								
site 1	0	0	0	0	0	0	0	0
site 2	0	0	0	0	0	0	0	0
site 3	0	0	0	0	0	0	0	0
site 4	0	0	0	0	0	0	0	0
site 5	0	0	0	0	0	0	0	0
Histamine								
site 1	0	0	2	3	2	3	1	3
site 2	0	0	2	3	1	3	2	3
site 3	0	0	2	3	2	3	2	3
site 4	0	0	1	1	1	2	2	3
site 5	0	0	2	2	1	2	2	3
Non-polar vehicle								
site 1	0	0	0	0	0	0	0	0
site 2	0	0	0	0	0	0	0	0
site 3	0	0	0	0	0	0	0	0
site 4	0	0	0	0	0	0	0	0
site 5	0	0	0	0	0	0	0	0

Table S11. Body weights (g) of the mice applied with CBS-400 extract, vehicle and positive control.

**CBS-400 extract**						
**Animal #**	**Day 0**	**Day 1**	**Day 2**	**Day 3**	**Day 4**	Day 5
1	19.3	20	20.4	20.6	20.2	19.2
2	19.8	21.2	21.5	21.3	21.6	21
3	20.8	20.9	18.8	19.3	19.3	19.2
4	20.8	21.4	19.8	19.8	19.7	19.6
5	20.4	20.9	21	20.6	20.5	20.8
Vehicle						
1	20.5	20.2	20.3	20.8	21	20.7
2	19.9	20.2	19.9	20.6	20.2	20.2
3	22.5	22.5	20.4	22.6	22.9	22.5
4	19.4	19.8	22.9	20	20	20
5	20.4	20.5	20.7	21	20.6	20.3
2,4-dinitrochlorobenzene (positive control)						
1	20.6	20	19.5	19.9	20.1	19.8
2	18.6	19.9	19.7	19.1	18.7	19.8
3	21.2	22.6	21.7	21.9	20.8	20.4
4	18.3	19.5	19.8	19.8	18.8	19.2
5	19.3	20.7	20.5	20.2	19.3	19.6

Table S12. Weights (mg) of the lymph nodes of the mice applied with CBS-400 extract, vehicle and positive control.

**Animal #**	**CBS-400 Extract**	**Vehicle**	**2,4-dinitrochlorobenzene (Positive Control)**
1	7.4	3.8	23.5
2	3.8	5.1	21.6
3	5.1	3.9	22.8
4	5.5	5.7	24.6
5	3.9	5.1	17.4

Table S13. Absorbance (O.D. value) of the lymph node suspension of the mice applied with CBS-400 extract, vehicle and positive control at 450 and 630 nm.

**Animal #**	**Blank**	**CBS-400 Extract**	**Vehicle**	**2,4-dinitrochlorobenzene (Positive Control)**
		450 nm		
1	0.099	0.263	0.124	0.480
2	0.141	0.343	0.296	0.633
3	0.109	0.294	0.235	0.553
4	0.139	0.285	0.229	0.539
		630 nm		
1	0.05	0.058	0.048	0.048
2	0.05	0.051	0.047	0.045
3	0.044	0.056	0.045	0.048
4	0.046	0.053	0.046	0.043

Table S14. Area ratios of residual CBS-400 implant and new bone at 3D, 4W, 8W and 12W post-operation for implantation group and area ratio of native bone at 12W for blank.

	**Implantation Group**								**Blank**	
	**3D**		**4W**		**8W**		**12W**		**12W**	
	**4 mm**	**5 mm**	**4 mm**	**5 mm**	**4 mm**	**5 mm**	**4 mm**	**5 mm**	**4 mm**	**5 mm**
Sample	Residual implant ratio (%)									
1	33.1	26.69	10.68	6.31	8.40	5.46	2.17	1.49		
2	32.5	24.72	26.02	21.54	14.10	11.74	11.42	9.45		
3	31.25	24.74	12.08	7.00	12.42	8.26	8.11	5.60		
4			14.37	10.01	5.74	3.77	3.91	2.82		
5			15.30	10.72	3.35	2.53	4.26	3.11		
6			14.75	11.05	5.71	3.65	0.40	0.26		
Sample	New bone formation ratio (%)								Native bone area ratio (%)	
1			47.13	47.15	47.86	44.46	17.41	14.85	22.13	23.28
2			28.87	33.85	35.23	37.30	43.96	41.46	35.98	36.90
3			49.19	50.14	43.72	47.17	41.75	39.75	11.58	15.12
4			39.17	41.56	39.82	39.39	31.48	32.77	22.21	21.01
5			42.21	38.73	25.93	28.85	23.83	25.31	30.19	27.37
6			47.89	48.37	31.34	39.09	23.84	25.68	17.87	21.45

© 2020 by the authors. Submitted for possible open access publication under the terms and conditions of the Creative Commons Attribution (CC BY) license (http://creativecommons.org/licenses/by/4.0/).
